# The Challenges of The Diagnostic and Therapeutic Approach of Patients with Infectious Pathology in Emergency Medicine

**DOI:** 10.3390/jpm14010046

**Published:** 2023-12-29

**Authors:** Silvia Ioana Musuroi, Adela Voinescu, Corina Musuroi, Luminita Mirela Baditoiu, Delia Muntean, Oana Izmendi, Romanita Jumanca, Monica Licker

**Affiliations:** 1 Doctoral School, “Victor Babeș” University of Medicine and Pharmacy, 300041 Timisoara, Romania; silvia.musuroi@umft.ro (S.I.M.);; 2 Internal Medicine Department, Municipal Emergency Clinical Hospital, 300254 Timisoara, Romania; 3 Microbiology Department, Multidisciplinary Research Center of Antimicrobial Resistance, “Victor Babes” University of Medicine and Pharmacy, 300041 Timisoara, Romania; muntean.delia@umft.ro (D.M.); licker.monica@umft.ro (M.L.); 4 Microbiology Laboratory, “Pius Brinzeu” County Clinical Emergency Hospital, 300723 Timisoara, Romania; 5 Epidemiology Department, “Victor Babes” University of Medicine and Pharmacy, 300041 Timisoara, Romania; baditoiu.luminita@umft.ro; 6 Romanian and Foreign Languages Department, “Victor Babes” University of Medicine and Pharmacy, 300041 Timisoara, Romania; romanita.jumanca@umft.ro

**Keywords:** *Pseudomonas*, *Acinetobacter*, antibiotic

## Abstract

The emergency department (ED) represents an important setting for addressing inappropriate antimicrobial prescribing practices because of the time constraints and the duration of microbiological diagnosis. The purpose of this study is to evaluate the etiology and antimicrobial resistance (AMR) pattern of the community-acquired pathogens, as well as the epidemiological characteristics of patients admitted through the ED, in order to guide appropriate antibiotic therapy. Methods: A retrospective observational study was performed on 657 patients, from whom clinical samples (urine, purulent secretions, blood cultures, etc.) were collected for microbiological diagnosis in the first 3 days after presentation in the ED. The identification of pathogens and the antimicrobial susceptibility testing with minimum inhibitory concentration determination were carried out according to the laboratory protocols. Results: From the 767 biological samples analyzed, 903 microbial isolates were identified. *E. coli* was most frequently isolated (24.25%), followed by *Klebsiella* spp., *S. aureus* (SA), and non-fermentative Gram-negative bacilli. *E. coli* strains maintained their natural susceptibility to most antibiotics tested. In the case of *Pseudomonas* spp. and *Acinetobacter* spp., increased rates of AMR were identified. Also, 32.3% of SA strains were community-acquired MRSA. Conclusions: The introduction of rapid microbiological diagnostic methods in emergency medicine is imperative in order to timely identify AMR strains and improve therapeutic protocols.

## 1. Introduction

Antibiotic-resistant bacteria such as methicillin-resistant *S. aureus* (MRSA) or extended-spectrum beta-lactamase (ESBL) producing Gram-negative bacilli (GNB) have emerged and spread from the hospital into the community. Inappropriate antibiotic (AB) use in human and veterinary medicine is the most important preventable cause of antimicrobial resistance (AMR) in both hospital-acquired infection (HAI) and community-acquired infection (CAI). Infections with resistant pathogens are associated with increased morbidity, mortality, and costs, and in addition, represent an important patient safety issue [1,2].

Emergency departments (EDs) are found at the interface between community and hospital and are an important setting concerning the approach of inappropriate antimicrobial prescribing practices, given their frequent use in these areas. ED clinicians routinely prescribe antimicrobials to patients for a wide variety of infections: skin and soft tissue, urinary tract, bloodstream, upper and lower respiratory tract, etc.

Practitioners in this setting have the unique opportunity to have a positive impact on the management of AB in both inpatient and outpatient settings, with important implications for both sectors. There are, for example, observational studies conducted in the ED, reporting significant rates of AB overprescribing in acute bronchitis (over 75% of prescriptions being for broad-spectrum AB, despite a certain improvement in clinical status) [3]. Reducing unnecessary AB administration is imperative, not only for lowering AMR rates in the community, but also for individual patient safety, given the increased rate of allergic reactions and the development of secondary infections associated with antibiotic administration, such as the *C. difficile* infection [4,5].

The literature data underline the importance of correct AB management in the ED and provide practical recommendations drawn from the existing evidence concerning the application of different strategies and tools that could be implemented in the ED: development of clinical guidelines, clinical decision support systems, or implementation of rapid diagnostic methods [6,7].

Antimicrobial stewardship comprises a collection of strategies, policies, and guidelines that aim to provide training and evaluation and collectively result in the optimization of antibiotic prescribing practices. It has been found that when Antimicrobial Stewardship Programs (ASP) are effectively implemented and monitored, they provide a measurable impact across multiple clinical departments: reducing drug costs, duration of treatment, adverse events to antibiotics, and local resistance. However, to date, ASPs have been targeted primarily at the hospital setting and there is a lack of literature data on antimicrobial stewardship strategies in EDs [8,9].

The implementation of ASP in the ED represents a challenge due to the specific conditions of activity in this compartment, such as the large number of patients examined/24 h, the limited time and equipment to support a rapid diagnosis, the decision regarding admission or discharge, and treatment of the patient at home [10].

The purpose of this study was to evaluate the etiology and AMR pattern of the community-acquired pathogens, as well as the epidemiological characteristics of patients admitted through the ED, within the largest tertiary emergency hospital in the western part of the country, in order to guide appropriate antibiotic therapy.

## 2. Materials and Methods

A retrospective observational study was carried out in the Emergency Department (ED) of the “Pius Brînzeu“ Clinical County Emergency Hospital Timișoara (SCJUPBT), over a period of 6 months, from 1 January 2021 to 30 June 2021. This institution is a tertiary teaching hospital, affiliated to the university, with 1174 beds, providing medical care for the western region of Romania. The ED of SCJUPT, the largest and most representative in Western Romania, has 24 workstations (beds), with approximately 80,000 patients per year, an average length of stay of about 4 h, and an admission rate of about 23%.

In accordance with the criteria of the Centers for Disease Control and Prevention, an infection presented on admission to the hospital or developing within 48 h or less from the time of admission was defined as community-acquired [11,12].

Following this definition, the inclusion criteria for CAI-ED patients were as follows:

- The diagnosis of the infection was based on the results of the microbiological tests, corroborated with the symptoms, the clinical examination, and the results of other paraclinical investigations recorded in the database and in the hospital documents.
- The infection was present at the time of ED presentation or within 48 h from hospital admission.
- Patients over 18 years old.

**Figure 1:**
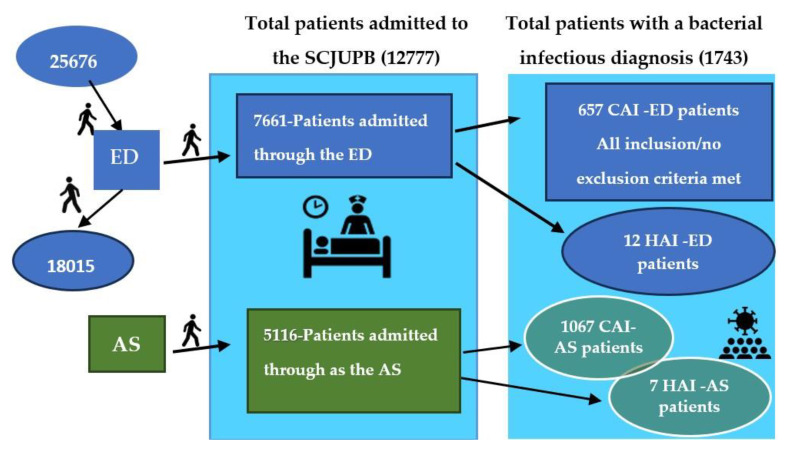
Study flow diagram. Legend: ED—emergency department, AS—admissions service, CAI—community-acquired infection, HAI—hospital-acquired infection.

The following exclusion criteria have been used: patients with a negative microbiological result (C1), colonized, not infected patients (C2), patients diagnosed with an infection after 3 days of hospitalization and those who presented in the ED with an infection but have been discharged for a maximum of 48 h from another hospital or are within 30 days postoperatively (90 days in case of implant) (C3), patients who come from chronic care or elderly care units (C4); 21 ED patients were excluded from the study based on these exclusion criteria (4-C1, 5-C2, 10-C3, 2-C4). Figure 1 presents the study flow diagram.

The samples for microbiological diagnostics were collected from patients in the ED, but also from clinical wards where they were transferred to and only from the hospitalized patients, not from those discharged from the ED.

Samples were taken before antibiotic administration, and if this was not possible, empirical antibiotic therapy was initiated, followed by de-escalation, or changing the antibiotic depending on the antimicrobial susceptibility testing (AST) result. Regarding patients with sepsis, the blood culture was collected in the first hour after admission to the ED, just before the administration of the first dose of antibiotic. If this was not possible, BD BACTEC™ Plus Aerobic medium (Becton, Dickinson and Company, 7 Loveton Circle, Sparks, USA) was used, which contains resins for antibiotic neutralization.

Pathogen identification, AST, and minimum inhibitory concentration (MIC) were performed according to the protocols of the Microbiology Laboratory, using MALDI-TOF-Bruker and VITEK^®^ 2 systems (Compact 60, BioMérieux, Marcy, l’Etoile, France). AST interpretation was performed according to CLSI standards [13].

For the clinically significant bacteria, depending on their acquired antibiotic resistance phenotypes, according to the CLSI standard, as well as the classifications of Magiorakos, Kadri, and other researchers, the following classifications have been used [13,14,15,16,17,18]:

- Methicillin-resistant *S. aureus* (MRSA): *S. aureus* with MIC ≥ 4 to oxacillin [14].
- Multidrug-resistant (MDR) bacteria: with resistance to at least one antibiotic from three or more classes of antibiotics active for a given species [15].
- Extensively drug-resistant bacteria (XDR): with resistance to at least one agent from all antimicrobial classes except one or two classes [15].
- Extended-spectrum beta-lactamase (ESBL) secreting Gram-negative bacilli (GNB): with resistance to all penicillins/cephalosporins [13,16].
- Carbapenem-resistant GNB (CR-GNB): enterobacteria with MIC ≥ 4 to imipenem, meropenem, and non-fermentative GNB with MIC ≥ 8 to imipenem, meropenem [13,17].
- Difficult-to-treat resistance (DTR): bacteria resistant to all first-line antibiotics, represented by: carbapenems (imipenem, meropenem, ertapenem/doripenem), extended-spectrum cephalosporins (those relevant to the respective pathogens), fluoroquinolones (ciprofloxacin, levofloxacin, moxifloxacin) [18].

Statistical analysis of the data was performed using the EPI INFO version 7.2.50 (CDC, Atlanta, GA, USA). Numerical variables were defined by median and interquartile range (IQR) and category variables were defined by value, percentage, and CI 95%. The category-type variables were compared with the 2 × 2 contingency tables and the application of the hi^2^ test (Fisher exact test). The tests were two-tailed and the threshold value was set at *p* < 0.05.

## 3. Results

During the period under study, a total of 25,676 patients were presented to the ED, out of which 7661 (29.83%) were admitted through the ED. The total number of patients admitted to the SCJUPB was 12,777, so patients admitted through the ED accounted for 59.96% of the total admissions.

Evaluation of the proportion of transfer wards of ED admissions showed that the most requested specialties were Surgery (SUR), Neurology (NEUR), and Gastroenterology (GE) (16.88%/14.25%/10.31%), followed by Vascular Surgery (VS), Cardiology (CD), and Urology (URO) (Table 1).

Regarding the share of ED admissions compared to total hospital admissions, this was almost 100% for Cardiology (CD) (99.54%), Vascular Surgery (VS) (99.54%), and Nephrology (NEF) (99.54%) and over 80% for Neurology (NEUR) (99.54%), Neurosurgery (NSUR) (99.54%), Gastroenterology (GE) (84.67%), and Diabetes and Nutrition (DN) (83.25%), respectively.

From the study group of 7661 ED inpatients, 657 (8.57%) were diagnosed with CAI, causing, or associated with inpatient illness, for which treatment was instituted according to microbiological diagnosis and AST. ED patients with an infectious diagnosis accounted for 37.69% of all patients with bacterial infections admitted during this period (ED, AS, CAI, and HAI) (Figure 1). Table 2 presents the demographic and comorbidity characteristics of the ED-CAI patients.

**Figure 2:**
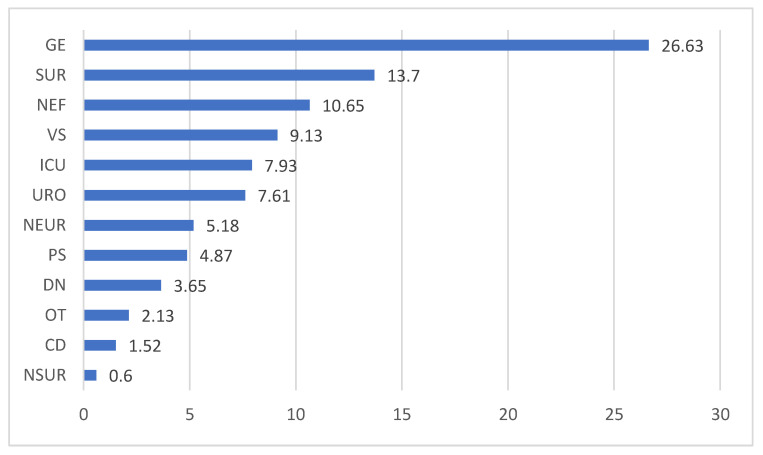
Breakdown by ward of CAI admitted through the ED (%). Legend: GE—Gastroenterology, SUR—Surgery, NEF—Nephrology, VS—Vascular Surgery, ICU—Intensive Care Unit, URO—Urology, NEUR—Neurology, PS—Plastic Surgery, DN—Diabetes and Nutrition, OT—Orthopedics-Traumatology, CD—Cardiology, NSUR—Neurosurgery.

In terms of the distribution of ED-CAI admissions by ward, the data obtained showed that Gastroenterology (GE) was the most requested ward for ED admissions, hospitalizing more than ¼ of the total of this group of patients (26.79%). These patients were admitted with a diagnosis of GI infection—angiocolitis (26.13%), pancreatitis (15.34%), or for an acute infectious complication of an underlying chronic GI disease—liver neoplasm (20.45%), pancreatitis (15.34%). At a distance, registering half of the GE frequencies was Surgery (13.7%), followed by Nephrology (10.65%) (Figure 2).

Surgery admissions were indicated for diagnosis of abscess/phlegmon/gangrene/ plague (36.67%), peritonitis (21.12%), appendicitis (15.56%), and intestinal occlusions (7.78%), while on the Nephrology ward, patients were transferred for sepsis with renal origine (48.57%), chronic kidney disease (47.14%), acute kidney injury (34.28%), and acute pyelonephritis (18.57%).

A special group of ED inpatients was the group of burned patients (N = 28) and the patients with trauma of various etiologies (N = 67), road traffic accidents, accidents at work, or domestic accidents.

Out of the group of patients with burns, 54% (N = 15) were major burns, transferred to the Functional Burns Unit (FBU), while 46% (N = 13) were with limited injuries, admitted to the Plastic Surgery ward (PS). Burned patients accounted for 0.36% of all ED admissions. Burned patients with infected burn injuries (23) represented 3.5% of all ED-CAI patients being admitted to the PS (60%) and FBU (40%) wards.

In terms of age decade distribution, of the 657 ED-CAI patients, 80.42% (456 patients) were in the 50–80 age decade. The study showed that women’s referral to ED services increases from age 50 onwards and peaks in the 70–79 age range. For men, referral increases from 40 years of age and peaks between 60 and 69 years of age.

**Figure 3:**
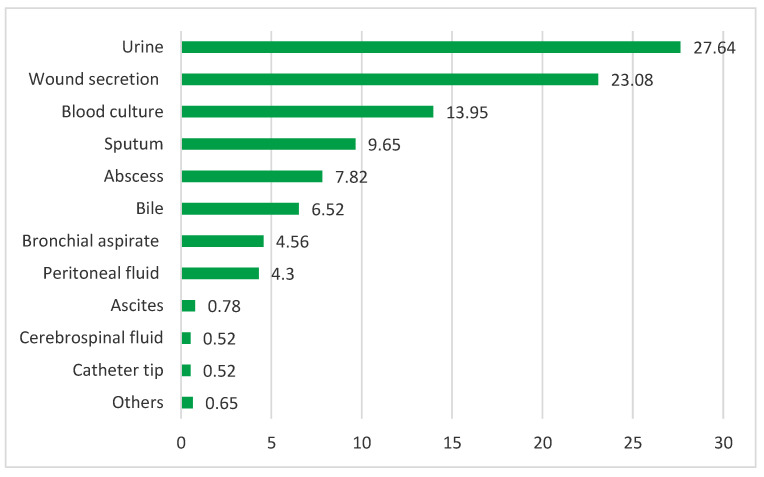
Clinical samples collected from ED-CAI patients (%).

Regarding the study of ED-CAI infections, from 657 ED-CAI patients, 767 clinical samples were collected for bacteriological diagnosis. The most numerous were urine cultures, wound drainage, and blood cultures (27.64%, 23.08%, 13.95%). Internal fluids together account for a significant percentage of 11.60% (Figure 3).

Out of the 767 samples analyzed, 903 microbial isolates were identified, of which 48.95% were *Enterobacterales*, 10.08% non-fermentative GNB, 34.22% Gram-positive cocci (GPC), and 4.98% fungi. The bacterial species most represented were *E. coli*, accounting for approximately ¼ of the total strains isolated (24.25%), followed by *Klebsiella* spp. (13.73%) and *S. aureus* (10.63%) (Table 3).

Table 4 and Table 5 show the distribution of isolates in clinical samples and the distribution of AMR phenotypes.

Nearly 50% of the identified *E. coli* strains (47.03%) were from urine cultures, followed by wound drainage (12.32%) and internal fluids: bile fluid, peritoneal fluid, blood cultures (9.59%, 8.68%, 7.76%) (Table 4). In terms of AMR, 25% of *E. coli* strains showed resistance to trimethoprim/sulfamethoxazole (SXT) and 17.8% showed resistance to fluoroquinolones (FQ); 6.4% recorded ESBL phenotype and 9% fell into the MDR category (Table 5).

The second most common isolate in ED-CAI was *Klebsiella* spp. The highest number of *Klebsiella* isolates was in urine cultures (24.20%), followed by sputum (16.93%) and abscess cultures (10.48%) (Table 4). AMR was marked by the identification of ESBL (11.30%) and CR (7.28%) strains, respectively, SXT- and FQ-resistant strains (14.51% and15.32%). As a result, 12.09% of strains were MDR type, 7.28% DTR type, and 2.50% were XDR strains (Table 5).

*Klebsiella* spp. were highlighted by the statistically significant higher frequency of the phenotypes: XDR (*p* = 0.046), DTR (*p* < 0.001), CRE (*p* < 0.001), and R-SXT (*p* < 0.001) vs. those of *E. coli*. ESBL isolates or strains with resistance to aminoglycosides, respectively, and resistance to fluoroquinolones, were identified in similar proportions (*p* = 0.149/0.177/0.653), as well as MDR strains (*p* = 0.849).

Non-fermentative GNB species had a low representation, with a frequency of 6.87% for *Pseudomonas* spp. and 1.88% for *Acinetobacter* spp., respectively (Table 3).

*Pseudomonas* spp. had the highest frequencies in cultures from wound drainage (40.32%), followed by cultures from bile fluids (12.9%) and urine cultures (12.90%) (Table 4); 19.35% were CR-resistant strains, 17.74% showed resistance to FQ, and 11.29% resistance to AG; 24.19% of strains were identified in the MDR and 11.29% in the DTR category, while the frequency of strains with extremely limited therapeutic options (XDR) was 6.45% (Table 5).

*Acinetobacter* spp. had the highest frequencies in cultures from wound drainage (58.83%). The remaining strains were present in bronchial aspirates and peritoneal fluid samples (Table 4). AMR studies reported high incidences of acquired resistance phenotypes of *Acinetobacter* spp. strains: over 50% CR and AG resistance phenotype. Accordingly, 52.94% of isolates fell into the DTR, 58.82% in the MDR category, and 23.15% were XDR strains (Table 5).

Among non-fermenters, *Acinetobacter* spp. isolates were statistically significant more resistant vs. those of *Pseudomonas* spp., both for the phenotypes: DTR (*p* < 0.001), MDR/ESBL (*p* = 0.004), CRE (*p* = 0.011), R-AG (*p* < 0.001), and R-FQ (*p* = 0.022). Only the XDR strains had similar percentages (*p* = 0.060).

Among the GPC, the highest frequency was recorded by *Staphylococcus aureus* (SA) strains. The majority of SA-positive cultures were taken from wounds (53.12%), followed by sputum and bronchial aspirates (10.42%, 10.42%) (Table 4). Phenotypic analysis showed that SA strains from CAI were resistant to antibiotics commonly used for the treatment of these infections. Thus 52% were beta-lactamase-producing strains, 32.3% were MRSA and 30.2% were identified with macrolide-lincosamide-streptogramin (MLSB) resistance phenotype, respectively. Consequently, the frequency of MDR strains was 37.5%. Penicillin resistance of *S. aureus* was significantly higher versus enterococci (*p* < 0.001) and macrolide resistance did not differ (*p* = 1.00).

## 4. Discussion

The present study was conducted during January–June 2021, namely, 181 days. The year 2021 was a pandemic year, located in the middle of the interval dominated by the SARS CoV-2 infection (February 2020–March 2022), in which hospital admissions were carried out according to well-established protocols, with limitations aimed at preventing intra-hospital transmission of the virus. The SCJUPBT-ED was requested by an average of 142 patients/day (25,676/181 days), of which, an average of 42 patients/day were admitted, out of an average total of 71 admissions/day/hospital, a flow that represented a percentage of approximately 60% ED admissions out of total admissions in the entire hospital.

The pathology of ED-CAI patients was split between surgical and medical specialties, but surgical pathology was noted to have a higher frequency of cases than medical pathology (53.64% versus 46.35%). This distribution is explained by the fact that the study covered the first 6 months of the second pandemic year 2021. In that year, the addressability of patients to tertiary medical units was strongly influenced by the restrictions imposed by the epidemiological situation, with the prioritization and admission of severe cases and medical/surgical emergencies. The hospitalization ward indirectly reflects the prevalent types of severe pathologies, which required hospitalization and immediate treatment, despite the pandemic context.

Most of the antibiotic treatments initiated in the ED for these patients are empirical. Antibiotic administration is profoundly influenced by patient demographic variables as well as diagnosis. High prescription rate use of antimicrobial treatments, regardless of these variables, however, has been observed in ED low-resource settings, highlighting the importance of surveillance in order to implement targeted intervention [19,20]. Moreover, in countries with a high consumption of antibiotics in Europe (such as Romania), an increase in antibiotic consumption is observed in winter and spring, thus causing a selective pressure and subsequently, an increase in multidrug resistance in community bacterial strains after a time delay of several months. These seasonal variations were also noticed by other researchers [21].

In our ED, antibiotics are administered only in cases of suspicion of sepsis, in the first hour after admission, immediately after having taken the samples intended for the microbiological examination, in accordance with the suspected source of sepsis. To medical practice, it is important that blood culture positivity is reduced by antimicrobial therapy, but remains high after a single dose of antibiotics, as shown in recent studies [22].

In this study, the majority of ED-CAI patients were over 50 years old, with approximately 55% falling within the 60–79 age range, consistent with findings from other studies [23,24]. ED-CAI patients over 80 years old were lower in number (15%) and they represented more than 2/3 of all admitted patients. The gender distribution of ED patients was not significant; however, the need for hospital medical care was significantly higher in men than in women for the age group 60–69 years (*p* = 0.043).

The strategy for dealing with the ED-CAI infectious patient depends on the framing of the disease—medical or surgical pathology. In the present study, ED-CAI patients with surgical pathology were 7.29% more common than those with medical diagnosis. It was noted that surgical patients (SUR+VS) accounted for about 23% of all infection admissions and renal patients (NEF+URO) achieved a significant frequency of 18.26%.

The problem of burned and trauma patients is a great challenge for the ED and transfer wards (BFU, respectively, ICU, OT, NSUR, PS), both in terms of medical treatment, length of stay, and cost of care. From the 28 burned patients admitted through the ED, 294 positive bacterial cultures were recorded throughout the admission, which means an average of 10.50 positive samples/patient and an average length of stay of 30 days, with a maximum of 156 days in isolation [25]. These figures show the importance of sampling for the diagnosis of microbial biofilms in these infections, as the biofilm is known to delay the healing even with optimal treatment instituted early [26,27].

The same issue of biofilm detection is posed for all types of wounds and catheter tip cultures, but also for respiratory tract cultures (sputum and bronchial aspirate). The most common bacterial species in wound biofilms (burn, ischemic, gangrenous) are *S. aureus* [28,29] and *P. aeruginosa* [30,31] with AMR to local and general treatments. *Pseudomonas* spp. are known to form biofilms in secretions of pulmonary patients, especially those with cystic fibrosis. Consequently, in these cases, genotypic investigation and biofilmography should be viewed as routine investigations for the institution of treatment.

The implementation of ASP-ED strategies for the management of CAI patients must take into consideration the peculiarities of ED functioning—rapid treatment decision-making in the context of time-constrained bacteriological investigation and access to rapid diagnostic tools. In this regard, Nauclér [32] has illustrated that rapid initiation of antibiotic treatment is crucial for patients with severe infections. He concluded that the literature supports prompt administration of effective antibiotics for septic shock and bacterial meningitis, but there is no clear evidence that delayed initiation of therapy is associated with a worse outcome for less severe infectious syndromes. For patients in whom bacterial infections are suspected, suspending antibiotic therapy until microbiological diagnostic results are available (e.g., up to 4–8 h) seems acceptable in most cases, except in the situation where septic shock or bacterial meningitis is suspected. This approach promotes the use of ecologically favorable antibiotics in the ED, reducing the risks of side effects and resistance selection.

To initiate an AB treatment in the ED, May [7] shows that the most important questions related to AMS to be answered at this stage are: “Is there a rationale for starting antimicrobial treatment for this diagnosis? Should I treat now? Or can I wait?” Currently, the answer to initiating antibiotic therapy in the ED is facilitated by the provision of rapid diagnostics through molecular techniques for the diagnosis of viral infections and the presence of biomarkers needed to differentiate bacterial from viral infections. Incorporating these findings into the clinical diagnostic process in the ED has the potential to significantly reduce unnecessary antibiotic use [10,33,34].

The use of procalcitonin as a biomarker has been considered when decision-making in chronic lung disease. There are studies that have shown a reduction in total antibiotic exposure (days of administration) and adverse effects based on values of this biomarker [35,36].

Multiplex PCR investigation on platforms such as rapid syndromic testing is another method that would allow rapid identification of pathogens and detection of resistance genes. Thus, Sun L. (2021) [37] showed in his study the overall sensitivity and specificity of Unyvero at detecting bacteria in lower respiratory tract infections was high (84.0% and 98.0%). The overall concordance between Unyvero and routine culture was 69/84 (82.1%). In addition, Unyvero showed good performance for antibiotic-resistant bacteria, except for *Pseudomonas aeruginosa*.

Similar agreements were published by Collins et al. [38], who compared the performance of the Unyvero LRT panel with routine bacterial culture methods on 175 bronchoalveolar lavage (BAL) samples and reported a sensitivity of 96.5% and a specificity of 99.6% among microbial targets. For antibiotic resistance markers in the LRT BAL lower respiratory tract panel, a positive predictive value (PPV) of 100% was reported. In another recent publication, Pickens et al. [39] reported a sensitivity of 85.7% and a specificity of 98.4% for 620 respiratory samples (395 bronchoscopic or none bronchoscopic BAL samples, 225 aspirates) using the Unyvero LRT panel.

Regarding GNB resistance genes, Klein [40] showed that the detection of a resistance gene does not necessarily link it to the host bacteria. However, for GNB, there were strong genotypic and phenotypic correlations of Unyvero results with the corresponding isolates. The reporting of resistance genes may provide a clue over the presence of an underlying resistant organism, which may have implications regarding infection prevention and control (e.g., if blaKPC, blaNDM, blaVIM, or blaOXA-48 is detected), even if the species with which the gene is associated is unknown.

Such an approach was adopted for some patients in the present study, so that multiplex PCR investigation on the Unyvero platform of an ED-CAI patient with polytrauma identified in blood culture the association of *A. baumannii* and *P. aeruginosa*, with the presence of NDM, OXA-24, SUL1, gyrA83 Pseu resistance genes, and of an ED-CAI patient transferred in GE, Unyvero investigation of a bronchial aspirate identified *A. baumannii* and *P. aeruginosa* and SUL1, gyrA83 Pseu resistance genes.

In the present study, the identification of germs responsible for ED-CAI infections has shown that the most frequently isolated strains were *E. coli* (24.25%), followed by *Klebsiella* species (13.73%) and *S. aureus* (10.63%). Benkő [41] showed in the ED study conducted in 2020 that she identified germs in the same hierarchy (*E. coli* (44.10%), *Klebsiella* spp. (13.40%), *S. aureus* (11.30%)), with the caveat that in this study, *E. coli* isolates had almost double the frequency, tending to account for half of the total number of strains.

Most ED-CAI patients with *E. coli* strains were admitted to GE wards (31.96%), kidney disease wards (NEF+URO, 21.46%), and SUR wards (18.26%). Isolates were identified in urine cultures and internal fluids (biliary, peritoneal, blood) and posed no AMR problems, with a small number being MDR, respectively, DTR strains. However, resistance to sulfonamides (R-S) and fluoroquinolones (R-FQ) was noted, which argues for the frequent use of these antibiotics in community infectious pathology [42] and draws attention to their use in the empiric therapy in ED.

*Klebsiella* spp. (13.73%) were isolated from urine cultures, RT cultures (sputum and bronchial aspirate), and wounds/abscesses of ED-CAI patients transferred to the GE, NEF+URO, SUR, and ICU wards (35.92%, 23.30%, 14.56%, and 11.65%). *Klebsiella* strains showed a higher percentage of MDR (12.9%), with acquired beta-lactam resistance phenotypes (ESBL and CR) and high frequencies of R-FQ, a fact also presented in other studies [10]. The difficult problem of establishing treatment regards the XDR strains (2.5%), with treatment options limited to, at most, two classes of antibiotics, which were identified in GE and SUR patients.

As for *S. aureus* strains, they were mostly identified in wound samples, respectively, RT samples of patients who were transferred to SUR, GE, NEF+URO, and ICU wards (48.14%, 14.81%, 11.12%, and 8.64%). The incidence of SA-MDR was high, (37.5%), explained by the frequency of MRSA and MLSB strains. A much lower rate of MRSA (only 0.4%) was identified in a study that set out to determine AMR in microorganisms causing community-onset bacteremia [43].

Non-fermentative GNBs were represented by *Pseudomonas* spp. and *Acinetobacter* spp., known to have high AMR behavior in hospital environments [44,45,46].

*Pseudomonas* spp. were reduced in number (6.87%), being mainly identified in wound drainage, RT samples (bronchial aspirates and sputum), and internal fluids (biliary and peritoneal). Approximately ¼ of these strains were MDR, with high frequencies of CR and FQ resistance phenotypes (almost 20% of them). Samples were from ED-CAI patients with transfer diagnoses for GE, SUR, and NEF+URO (30.07%, 30.18%, and 18.86%).

*Acinetobacter* spp. were present in low numbers, in samples from ED-CAI patients. The majority were identified in wound drainage of burned patients, patients with lower limb pathology (ischemia, gangrene), and infected traumatic injuries, 81.25% being transferred to surgical wards (VS, PS) and FBU. The problem raised by these isolates, however, is the high degree of AMR, with a frequency of almost ¼ of their number of XDR strains. The high resistance to carbapenems, the only effective beta-lactams on *Acinetobacter* species (52.94%), together with resistance to AG and FQ (52.94%, 47.05%) made infections caused by these strains extremely difficult to treat, due to the lack of effective therapy.

The current study brings more information about CAI patients hospitalized by the ED, a topic that is not often addressed in the literature, at least in our geographical area, where there is little data in this regard.

Moreover, because of the AMR studies published by our team and in accordance with the Stanford Antimicrobial Safety & Sustainability Program 2019 [47], the SCJUPBT Antibiotic Prophylaxis Guide has been updated.

Also, our recommendations regarding the purchase and installation of an Unyvero equipment in the ED, for the rapid molecular identification of MDR pathogens, as well as the screening of patients to identify colonization with these organisms in the ED, were discussed with the hospital management.

However, the current study has some limitations. It addresses infectious pathology hospitalized through a single ED, and a single hospital, which is a reason why its generalizability may be limited. The study was conducted in a particular pandemic period, burdened by restrictions imposed by the health system, but also by the decreasing addressability of the population. The descriptive design did not allow clear evidence of these differences. Also, as with any retrospective study, there are potential biases such as selection and information bias.

## 5. Conclusions

During the period under study, the proportion of ED admissions increased, with a significant percentage of patients with infectious diagnoses, mostly elderly, with associated pathologies, hospitalized mainly in surgical wards. It was found that the disease state and the need for hospital care in men was earlier than in women.

Of the identified pathogens, GNB were mostly predominant. *E. coli* was isolated most frequently, but with maintenance of susceptibility to the usual ABs, while non-fermenters were isolated less frequently, but with increased AMR rates. In the case of SA strains, we noted the significantly increased percentage of community-acquired MRSA strains.

The ongoing study of AMR in ED isolates, as well as the introduction of rapid microbiological diagnostic methods are imperative to timely identify MDR strains and improve therapeutic protocols. We also emphasize the need for ASP in the ED with the identification of interventions to improve patient outcomes and care and reduce the consequences of antimicrobial use in the hospital and community.

## Figures and Tables

**Table 1:** The share of patients admitted through the ED on different wards of SCJUPBT.

ED Admissions by Ward of Total ED Admissions (%), January–June 2021					
Ward	No.	%	Ward	No.	%
Surgery (SUR)	1293	16.88	Orthopedics-Traumatology (OT)	629	8.21
Neurology (NEUR)	1092	14.25	Nephrology (NEF)	469	6.12
Gastroenterology (GE)	790	10.31	Neurosurgery (NSUR)	542	5.63
Vascular Surgery (VS)	717	9.36	Diabetes and Nutrition (DN)	338	4.41
Cardiology (CD)	652	8.51	Plastic and Reconstructive Surgery (PS)	311	4.06
Urology (URO)	623	8.13	Other	205	4.13
Total 7661 (100%)					

**Table 2:** Demographic and comorbidity characteristics of the cohort (657 patients).

Variable	N = 657	95% CI
M (n(%))	355 (54.03)	50.21–57.81
F (n (%))	302 (45.97)	42.19–49.79
Average age (average (IQR))	62.38 (53–73)	/
Comorbid conditions		
Peripheral artery disease (n (%))	61 (9.28)	7.30–11.75
Hypertensive patients (n (%))	112 (17.04)	14.37–20.11
SARS-CoV2 (n (%))	106 (16.13)	13.52–19.14
Diabetes mellitus (n (%))	69 (10.5)	8.38–13.08
Coronary artery disease (n (%))	43 (6.54)	4.90–8.70
Ischemic stroke (n (%))	41 (6.24)	4.63–8.36
Kidney stones (n (%))	9 (1.37)	0.72–2.58
Chronic kidney disease (CKD) (n (%))	47 (7.15)	5.42–9.38
Acute kidney injury (AKI) (n (%))	39 (5.93)	4.37–8.01
Renal neoplasia (n (%))	7 (1.06)	0.52–2.18
Cirrhosis (n (%))	61 (9.28)	7.30–11.75
Biliary lithiasis (n (%))	50 (7.61)	5.82–9.89
GI tract neoplasia (n (%))	53 (8.06)	6.22–10.40

Legend: GI—gastrointestinal.

**Table 3:** Proportion of microbial isolates identified from ED-CAI patients (% from 903 isolates).

903 Isolates	EC	KB	SA	EnC	CNS	STR	PSE	CAN	PRO	EnB	AcB	CIT	Other
Nr.	219	124	96	85	79	49	62	45	37	22	17	17	51
% from 903	24.25	13.73	10.63	9.41	8.75	5.43	6.87	4.98	4.1	2.44	1.88	1.88	5.65

Legend: EC—*E. coli*, KB—*Klebsiella* spp., SA—*S. aureus*, EnC—*Enterococcus* spp., CNS—coagulase-negative staphylococci, STR—*Streptococcus* spp., PSE—*Pseudomonas* spp., CAN—*Candida* spp., PRO—*Proteus* spp., EnB—*Enterobacter* spp., AbC—*Acinetobacter* spp., CIT—*Citrobacter* spp.

**Table 4:** Distribution of microbial isolates in clinical samples (%).

Bacterial Species (n)	Urine (%)	Wound Drainage (%)	Blood Culture (%)	Sputum (%)	Bronchial Aspirate (%)	Abscess (%)	Bile (%)	Peritoneal Fluid (%)
*E. coli* (219)	47.03	12.32	7.76	3.2	2.28	7.76	9.59	8.68
*K.pneumoniae* (124)	24.2	8.87	7.26	16.93	7.25	10.48	6.45	0
*S. aureus* (96)	6.25	53.12	9.37	10.42	10.42	6.25	0	0
*Pseudomonas* spp. (62)	12.9	40.32	6.45	12.9	6.45	1.61	12.9	3.22
*Acinetobacter* spp. (17)	0	58.83	5.88	5.88	11.7	0	5.88	11.76

**Table 5:** Classification of the most important GNB into resistance phenotypes (%).

Bacterial Species	DTR	MDR	XDR	ESBL	CR-GNB	R-AG	R-FQ	R-SXT	R-TE
*E. coli (%)*	0.5	9	0	6.4	0.45	1.8	17.8	25.2	1.8
*Klebsiella* spp. *(%)*	7.28	12.09	2.50	11.30	7.28	4.84	15.32	14.51	2.5
*Pseudomonas* spp. *(%)*	11.29	24.19	6.45	/	19.35	11.29	17.74	/	/
*Acinetobacter* spp. *(%)*	52.94	58.82	23.50	/	52.94	52.94	47.05	52.94	/

## Data Availability

Data are contained within the article.

## References

[1] Han J.H., Kasahara K., Edelstein P.H., Bilker W.B., Lautenbach E. (2012). Risk factors for infection or colonization with CTX-M extended-spectrum-β-lactamase-positive *Escherichia coli*. Antimicrob. Agents Chemother..

[2] Moran G.J., Krishnadasan A., Gorwitz R.J., Fosheim G.E., McDougal L.K., Carey R.B., Talan D.A. (2006). Methicillin-resistant *S. aureus* infections among patients in the emergency department. N. Engl. J. Med..

[3] Kroening-Roche J.C., Soroudi A., Castillo E.M., Vilke G.M. (2012). Antibiotic and bronchodilator prescribing for acute bronchitis in the emergency department. J. Emerg. Med..

[4] Gonzales R., Camargo C.A., MacKenzie T., Kersey A.S., Maselli J., Levin S.K., McCulloch C.E., Metlay J.P. (2006). Antibiotic treatment of acute respiratory infections in acute care settings. Acad. Emerg. Med..

[5] Metlay J.P., Camargo C.A., MacKenzie T., McCulloch C., Maselli J., Levin S.K., Kersey A., Gonzales R. (2007). Cluster-randomized trial to improve antibiotic use for adults with acute respiratory infections treated in emergency departments. Ann. Emerg. Med..

[6] May L., Cosgrove S., L’Archeveque M., Talan D.A., Payne P., Jordan J., Rothman R.E. (2013). A call to action for antimicrobial stewardship in the emergency department: Approaches and strategies. Ann. Emerg. Med..

[7] May L., Martin-Quirós A., Ten Oever J., Hoogerwerf J., Schoffelen T., Schouten J. (2021). Antimicrobial stewardship in the emergency department: Characteristics and evidence for effectiveness of interventions. Clin. Microbiol. Infect..

[8] Lawton R.M., Fridkin S.K., Gaynes R.P., McGowan J.E. (2000). Practices to improve antimicrobial use at 47 US hospitals: The status of the 1997 SHEA/IDSA position paper recommendations. Society for Healthcare Epidemiology of America/Infectious Diseases Society of America. Infect. Control Hosp. Epidemiol..

[9] (2012). Policy statement on antimicrobial stewardship by the Society for Healthcare Epidemiology of America (SHEA), the Infectious Diseases Society of America (IDSA), and the Pediatric Infectious Diseases Society (PIDS). Infect. Control Hosp. Epidemiol..

[10] Bishop B.M. (2016). Antimicrobial Stewardship in the Emergency Department: Challenges, Opportunities, and a Call to Action for Pharmacists. J. Pharm. Pract..

[11] Garner J.S., Jarvis W.R., Emori T.G., Horan T.C., Hughes J.M. (1988). CDC definitions for nosocomial infections. Am. J. Infect. Control..

[12] Dabar G., Harmouche C., Salameh P., Jaber B.L., Jamaleddine G., Waked M., Yazbeck P. (2015). Community- and healthcare-associated infections in critically ill patients: A multicenter cohort study. Int. J. Infect. Dis..

[13] CLSI (2021). Performance Standards for Antimicrobial Susceptibility Testing, M100.

[14] Turner N.A., Sharma-Kuinkel B.K., Maskarinec S.A., Eichenberger E.M., Shah P.P., Carugati M., Holland T.L., Fowler V.G. (2019). Methicillin-resistant Staphylococcus aureus: An overview of basic and clinical research. Nat. Rev. Microbiol..

[15] Magiorakos A.-P., Srinivasan A., Carey R.B., Carmeli Y., Falagas M.E., Giske C.G., Harbarth S., Hindler J.F., Kahlmeter G., Olsson-Liljequist B. (2012). Multidrug-resistant, extensively drug-resistant and pandrug-resistant bacteria: An international expert proposal for interim standard definitions for acquired resistance. Clin. Microbiol. Infect..

[16] Pitout J.D.D., Laupland K.B. (2008). Extended-spectrum beta-lactamase-producing Enterobacteriaceae: An emerging public-health concern. Lancet Infect. Dis..

[17] Nordmann P., Naas T., Poirel L. (2011). Global spread of Carbapenemase-producing Enterobacteriaceae. Emerg. Infect. Dis..

[18] Kadri S.S., Adjemian J., Lai Y.L., Spaulding A.B., Ricotta E., Prevots D.R., Palmore T.N., Rhee C., Klompas M., Dekker J.P. (2018). Difficult-to-Treat Resistance in Gram-negative Bacteremia at 173 US Hospitals: Retrospective Cohort Analysis of Prevalence, Predictors, and Outcome of Resistance to All First-line Agents. Clin. Infect. Dis..

[19] Yi S., Ramachandran A., Epps L., Mayah A., Burkholder T.W., Jaung M.S., Haider A., Whesseh P., Shakpeh J., Enriquez K. (2022). Emergency department antimicrobial use in a low-resource setting: Results from a retrospective observational study at a referral hospital in Liberia. BMJ Open.

[20] Shankar P.R. (2009). Medicines use in primary care in developing and transitional countries: Fact book summarizing results from studies reported between 1990 and 2006. Bull. World Health Organ..

[21] Martínez P., Rosmalen J., Bustillos Huilca R., Natsch S., Mouton J., Verbon A. (2020). Trends, seasonality and the association between outpatient antibiotic use and antimicrobial resistance among urinary bacteria in the Netherlands. J. Antimicrob. Chemother..

[22] Zornitzki L., Anuk L., Frydman S., Morag-Koren N., Zahler D., Freund O., Biran R., Liron Y., Tau L., Tchebiner J.Z. (2023). Rate and predictors of blood culture positivity after antibiotic administration: A prospective single-center study. Infection.

[23] Hong S.-I., Kim J.-S., Kim Y.-J., Seo D.-W., Kang H., Kim S.J., Han K.S., Lee S.W., Kim W.Y. (2022). Characteristics of Patients Who Visited Emergency Department: A Nationwide Population-Based Study in South Korea (2016–2018). Int. J. Environ. Res. Public Health.

[24] Lee J.H., Park G.J., Kim S.C., Kim H., Lee S.W. (2020). Characteristics of frequent adult emergency department users: A Korean tertiary hospital observational study. Medicine.

[25] Licker M., Musuroi C., Muntean D., Crainiceanu Z. Updates in the management of multidrug-resistant bacterial infections in burn patients. Proceedings of the 16th National Conference on Microbiology and Epidemiology.

[26] Percival S.L., McCarty S.M., Lipsky B. (2015). Biofilms and Wounds: An Overview of the Evidence. Adv. Wound Care.

[27] Schultz G., Bjarnsholt T., James G.A., Leaper D.J., McBain A.J., Malone M., Stoodley P., Swanson T., Tachi M., Wolcott R.D. (2017). Consensus guidelines for the identification and treatment of biofilms in chronic nonhealing wounds. Wound Repair Regen..

[28] Di Lodovico S., Bacchetti T., D’ercole S., Covone S., Petrini M., Di Giulio M., Di Fermo P., Diban F., Ferretti G., Cellini L. (2022). Complex Chronic Wound Biofilms Are Inhibited in vitro by the Natural Extract of Capparis spinose. Front. Microbiol..

[29] Roy S., Santra S., Das A., Dixith S., Sinha M., Ghatak S., Ghosh N., Banerjee P., Khanna S., Mathew-Steiner S. (2020). Staphylococcus aureus Biofilm Infection Compromises Wound Healing by Causing Deficiencies in Granulation Tissue Collagen. Ann. Surg..

[30] Moreau-Marquis S., Stanton B.A., O’Toole G.A. (2008). Pseudomonas aeruginosa biofilm formation in the cystic fibrosis airway. Pulm. Pharmacol. Ther..

[31] Jennings L.K., Dreifus J.E., Reichhardt C., Storek K.M., Secor P.R., Wozniak D.J., Hisert K.B., Parsek M.R. (2021). Pseudomonas aeruginosa aggregates in cystic fibrosis sputum produce exopolysaccharides that likely impede current therapies. Cell Rep..

[32] Nauclér P., Huttner A., van Werkhoven C., Singer M., Tattevin P., Einav S., Tängdén T. (2021). Impact of time to antibiotic therapy on clinical outcome in patients with bacterial infections in the emergency department: Implications for antimicrobial stewardship. Clin. Microbiol. Infect..

[33] Pulia M., Redwood R., May L. (2018). Antimicrobial Stewardship in the Emergency Department. Emerg. Med. Clin. N. Am..

[34] Barlam T.F., Cosgrove S.E., Abbo L.M., MacDougall C., Schuetz A.N., Septimus E.J., Srinivasan A., Dellit T.H., Falck-Ytter Y.T., Fishman N.O. (2016). Implementing an Antibiotic Stewardship Program: Guidelines by the Infectious Diseases Society of America and the Society for Healthcare Epidemiology of America. Clin. Infect. Dis..

[35] Mathioudakis A.G., Chatzimavridou-Grigoriadou V., Corlateanu A., Vestbo J. (2017). Procalcitonin to guide antibiotic administration in COPD exacerbations: A meta-analysis. Eur. Respir. Rev..

[36] Schuetz P., Wirz Y., Sager R., Christ-Crain M., Stolz D., Tamm M., Bouadma L., Luyt C.E., Wolff M., Chastre J. (2017). Procalcitonin to initiate or discontinue antibiotics in acute respiratory tract infections. Cochrane Database Syst. Rev..

[37] Sun L., Li L., Du S., Liu Y., Cao B. (2021). An evaluation of the Unyvero pneumonia system for rapid detection of microorganisms and resistance markers of lower respiratory infections-a multicenter prospective study on ICU patients. Eur. J. Clin. Microbiol. Infect. Dis..

[38] Collins M.E., Popowitch E.B., Miller M.B. (2020). Evaluation of a Novel Multiplex PCR Panel Compared to Quantitative Bacterial Culture for Diagnosis of Lower Respiratory Tract Infections. J. Clin. Microbiol..

[39] Pickens C., Wunderink R.G., Qi C., Mopuru H., Donnelly H., Powell K., Sims M.D. (2020). A multiplex polymerase chain reaction assay for antibiotic stewardship in suspected pneumonia. Diagn. Microbiol. Infect. Dis..

[40] Klein M., Bacher J., Barth S., Atrzadeh F., Siebenhaller K., Ferreira I., Beisken S., Posch A.E., Carroll K.C., Wunderink R.G. (2021). Multicenter Evaluation of the Unyvero Platform for Testing Bronchoalveolar Lavage Fluid. J. Clin. Microbiol..

[41] Benkő R., Gajdács M., Matuz M., Bodó G., Lázár A., Hajdú E., Papfalvi E., Hannauer P., Erdélyi P., Pető Z. (2020). Prevalence and Antibiotic Resistance of ESKAPE Pathogens Isolated in the Emergency Department of a Tertiary Care Teaching Hospital in Hungary: A 5-Year Retrospective Survey. Antibiot.

[42] Grignon O., Montassier E., Corvec S., Lepelletier D., Hardouin J.-B., Caillon J., Batard E., EDBAC Study Group (2015). Escherichia coli antibiotic resistance in emergency departments. Do local resistance rates matter?. Eur. J. Clin. Microbiol. Infect. Dis..

[43] Rothe K., Wantia N., Spinner C.D., Schneider J., Lahmer T., Waschulzik B., Schmid R.M., Busch D.H., Katchanov J. (2019). Antimicrobial resistance of bacteraemia in the emergency department of a German university hospital (2013–2018): Potential carbapenem-sparing empiric treatment options in light of the new EUCAST recommendations. BMC Infect. Dis..

[44] El-Sokkary R., Uysal S., Erdem H., Kullar R., Pekok A.U., Amer F., Grgić S., Carevic B., El-Kholy A., Liskova A. (2021). Profiles of multidrug-resistant organisms among patients with bacteremia in intensive care units: An International ID-IRI survey. Eur. J. Clin. Microbiol. Infect. Dis..

[45] Baditoiu L., Axente C., Lungeanu D., Muntean D., Horhat F., Moldovan R., Hogea E., Bedreag O., Sandesc D., Licker M. (2017). Intensive care antibiotic consumption and resistance patterns: A cross-correlation analysis. Ann. Clin. Microbiol. Antimicrob..

[46] Axente C., Licker M., Moldovan R., Hogea E., Muntean D., Horhat F., Bedreag O., Sandesc D., Papurica M., Dugaesescu D. (2017). Antimicrobial consumption, costs and resistance patterns: A two year prospective study in a Romanian intensive care unit. BMC Infect. Dis..

[47] Stanford Health Care The SHC Antimicrobial Guidebook. https://med.stanford.edu/bugsanddrugs/guidebook.html.

